# The proprotein convertase PC5/6 is protective against intestinal tumorigenesis: *in vivo *mouse model

**DOI:** 10.1186/1476-4598-8-73

**Published:** 2009-09-08

**Authors:** Xiaowei Sun, Rachid Essalmani, Nabil G Seidah, Annik Prat

**Affiliations:** 1 Laboratory of Biochemical Neuroendocrinology, Clinical Research Institute of Montreal, affiliated to the University of Montreal, 110 Pine Avenue West, Montreal, Quebec, Canada

## Abstract

**Background:**

The secretory basic amino acid-specific proprotein convertases (PCs) have often been associated with cancer/metastasis. By controlling the cleavage of cancer-associated proteins, PCs play key roles in multiple steps of cancer development. Most analyses of the implication of PCs in cancer/metastasis relied on the use of *in vitro *overexpression systems or inhibitors that can affect more than one PC. Aside from the role of furin in salivary gland tumorigenesis, no other *in vivo *genetic model of PC-knockout was reported in relation to cancer development.

**Results:**

Since PC5/6 is highly expressed in the small intestine, the present study examined its *in vivo *role in intestinal tumorigenesis. Analysis of human intestinal tumors at various stages showed a systematic down-regulation of PC5/6 expression. Since gene inactivation of PC5/6 leads to lethality at birth, we generated mice lacking PC5/6 in enterocytes and analyzed the impact of the presence or absence of this PC in the mouse *Apc*^*Min*/+ ^model that develops numerous adenocarcinomas along the intestinal tract. This resulted in viable mice with almost no expression of PC5/6 in small intestine, but with no overt phenotype. The data showed that by themselves *Apc*^*Min*/+ ^tumors express lower levels of PC5/6 mRNA, and that the lack of PC5/6 in enterocytes results in a significantly higher tumor number in the duodenum, with a similar trend in other intestinal segments. Finally, the absence of PC5/6 is also associated with a premature mortality of *Apc*^*Min*/+ ^mice.

**Conclusion:**

Overall, these data suggest that intestinal PC5/6 is protective towards tumorigenesis, especially in mouse duodenum, and possibly in human colon.

## Background

Nine secretory proprotein convertases (PCs) of the subtilisin/kexin type (genes *PCSK1 *to *PCSK9*) were identified in mammals and are known as: PC1/3, PC2, furin, PC4, PC5/6, PACE4, PC7, SKI-1/S1P and PCSK9 [1,2]. The first 7 convertases cleave secretory precursor proteins at single or paired basic residues [2], whereas SKI-1/S1P [3] and PCSK9 [4] do not require a basic residue at the cleavage site. The basic amino acid (aa)-specific convertases process precursors of growth factors, receptors, polypeptide hormones, adhesion molecules, proteases, as well as cell surface proteins of infectious viruses and bacteria [2]. In some cases, furin and/or PC5/6 inactivate proteins such as endothelial and lipoprotein lipases [5], PCSK9 [6] and N-cadherin (Maret D. *et al*., *submitted*).

Overexpression of PC5/6, PACE4 and furin revealed that these proteinases can often cleave the same precursors, indicating a functional redundancy [6-12]. Evidence for *in vivo *redundancy was provided by furin inactivation in the liver, which revealed that most of the precursors analyzed were still processed, although to a lesser extent, in the absence of this ubiquitous convertase [13]. In contrast, *in vivo *studies demonstrated that in a spatio-temporal manner furin can uniquely process the Ac45 subunit of the vacuolar type H^+^-ATPase in pancreatic β-cells [14] and PC5/6 the TGFβ-like growth and differentiation factor Gdf11 in the developing embryo [15,16].

Various precursors cleaved by overexpressed furin, PC5/6, PACE4 and PC7 have been previously implicated in cancer and associated metastatic processes [17-19]. A correlation between the mRNA levels of some of these PCs and the degree of tumorigenicity has been reported [9,18-27]. Furthermore, injection/implantation of various cell lines expressing PC inhibitors, such as the antitrypsin derivative α1-PDX [9,12,20,24,27,28] or the inhibitory prodomain of PCs [26] suggested a critical role of the PCs in tumor growth and/or metastasis.

The convertase PC5/6 (previously known as PC5 or PC6) was characterized in 1993 and shown to be composed of two differentially spliced isoforms, a short 915 aa soluble PC5/6A [29], and a long membrane-bound 1877 aa PC5/6B [30]. In adult rodents, PC5/6 exhibits a wide tissue distribution [29], which in mice when analyzed by quantitative PCR (QPCR) revealed that the adrenal cortex and small intestine are the richest sources of PC5/6A and PC5/6B, respectively [31]. However, the function of PC5/6 in these tissues has not been addressed. PC5/6 can bind cell surface heparan sulfate proteoglycans and tissue inhibitors of metalloproteases *via *its C-terminal Cys-rich domain [32]. It also seems to differ from the other convertases in that it can get activated at the cell surface [1,33]. Knockout of the PC5/6 gene (*Pcsk5*) revealed that *Pcsk5*^-/- ^animals die at birth due to multiple malformations, including defects in antero-posterior patterning and heart formation [15,16]. Defective specification of segment identity, which leads to an increased number of thoracic and lumbar vertebrae and lack of tail, is likely due to the absence of processing of Gdf11 [15,16,34]. No obvious malformations were seen in the small intestine of *Pcsk5*^-/- ^embryos [15].

The specific role of PC5/6 in tumorigenesis/metastasis has not yet been investigated. PC5/6 expression was not detected in human breast, and generally not induced in breast cancer since it was present in only 2/30 tumors [35]. In contrast, its mRNA levels seem to correlate with tumor aggressiveness of head and neck- and lung tumor-derived cell lines [18], suggesting that PC5/6 may play a different role in metastasis compared to tumor growth. Whether this is related to its ability to process adhesion molecules [36], including the α-chain of various integrins [7,37] and N-cadherin (Maret D. *et al*., *submitted*) is not yet clear.

Colorectal cancer is the third most common form of cancer in the Western world. As a mouse model for this pathology, we used the *Apc*^*Min*/+ ^strain that harbors a heterozygote *Min *(*multiple intestinal neoplasia*) mutation in the *Apc (adenomatous polyposis coli) *gene. These mice spontaneously develop polyps all along the small intestine [38,39]. In order to assess the role of PC5/6 in intestinal tumorigenesis, we generated PC5/6 intestine-specific knockout mice (iKO) and crossed them with *Apc*^*Min*/+ ^mice. Our data show that mice carrying the *Min *mutation but lacking PC5/6 tend to exhibit a higher tumor number than *Apc*^*Min*/+ ^mice, especially in duodenum, and die significantly earlier.

## Methods

### Animals

Tg(Vil-cre) mice (stock number 004586) [40] and *Apc*^*Min*/+ ^mice (stock number 002020) [39] were from The Jackson Laboratory. Conditional knockout mice, in which the proximal promoter and exon 1 of *Pcsk5 *were flanked with *loxP *sites (*Pcsk5*^*flox*/*flox*^) [15], were crossed with Tg(Vil-cre) mice that express Cre under the control of the villin promoter. After two generations,*Pcsk5*^*flox*/*flox *^mice carrying (intestinal KO; iKO) or not (wild type; WT) one copy of the transgene were obtained and further intercrossed, yielding the F4 progeny used in this study, which exhibits a mixed background consisting of ~ 70% C57BL/6; 25% 129Sv and less than 5% SJL. When expressed, Cre leads to the recombination of the two *loxP *sites present in *Pcsk5*, resulting in the excision of ~ 3 kb of DNA including exon 1 (Δ1 alleles) and thereby gene inactivation.

### Tumor scoring in mouse intestine

Four month old mice were sacrificed by CO_2 _asphyxiation, and the whole intestine was immediately removed and rinsed with ice-cold PBS. The intestine was divided into duodenum, jejunum, ileum and colon. All sections were carefully split longitudinally, fixed in 8% paraformaldehyde, stained with 8% methylene blue and the tumors were counted under a binocular microscope.

### Quantitative RT-PCR

Tissue samples were dissected from PBS-rinsed intestine. Total RNA was extracted using Trizol reagent (Invitrogen), as recommended by the manufacturer. Typically, 250 ng of total RNA were used for cDNA synthesis in a total volume of 20 μL using SuperScript II reverse transcriptase, 25 μg/mL oligo(dT)_12-18_, 0.5 mM 2'-deoxynucleoside 5'-triphosphates, and 40 U of RNaseOUT, all products from Life Technologies, and used according to the recommendations of the manufacturer. cDNAs of human adenocarcinomas were purchased from Origene. The quantitative PCR (QPCR) was performed as previously described [41]. Specific primers (Table 1) were used for the simultaneous amplification of the normalizing cDNA for ribosomal protein S14 (human) or S16 (mouse), and the gene of interest.

**Table 1:** Sequences of primers used for QPCR

**Assessed mRNA**	**Forward Primer**	**Reverse Primer**
human PC5AB	ACTCTTCAGAGGGTGGCTA	GCTGGAACAGTTCTTGAATC

mouse PC5AB	TGACCACTCTTCAGAGAATGGATAC	GAGATACCCACTAGGGCAGC

mouse PC5A	AGGATTCAAGAACTGTTCCA	AGCATACAGAAGCCTCCTT

mouse PC5B	GCAATGCCTCCCACTCCC	TGCTCGTAAAACTCAGCCTCC

mouse Furin	CATGACTACTCTGCTGATGG	GAACGAGAGTGAACTTGGTC

Cre	ATGATCCGAATAACTACCTG	ACAATATTTACATTGGTCCAG

human S14	GGCAGACCGAGATGAATCCTCA	CAGGTCCAGGGGTCTTGGTCC

mouse S16	GCTACCAGGGCCTTTGAGATG	AGGAGCGATTTGCTGGTGTGG

### *In situ *hybridization

Mouse cRNA probes corresponding to the coding region for aa 20 to 348 of PC5/6 were synthesized using ^35^S-UTP and ^35^S-CTP (>1,000 Ci/mmol; Amersham Bioscience, Piscataway, NJ). Cryosections (8-10 μm) were fixed for 1 hour in 4% formaldehyde and hybridized overnight at 55°C as previously described [42]. For autoradiography, the sections were dipped in photographic emulsion (NTB-2, Kodak, Rochester, NY), exposed for 6-12 days, and developed in D19 solution (Kodak).

### PCNA immunohistochemistry

Tissues were fixed overnight in 4% paraformaldehyde at 4°C and embedded in paraffin. Proliferation cell nuclear antigen (PCNA) was visualized in sections of 6 μm thickness by incubation with a mouse antibody (1:50; Vector laboratories, Burlingame, CA) and a biotin-labeled secondary antibody (PerkinElmer, Boston, MA), and revelation with the Vectastain kit (Vector laboratories). Sections were also counterstained with hematoxylin and eosin.

## Results

### Expression of PC5/6 is lower in intestinal tumors versus adjacent normal tissues

**Figure 1:**
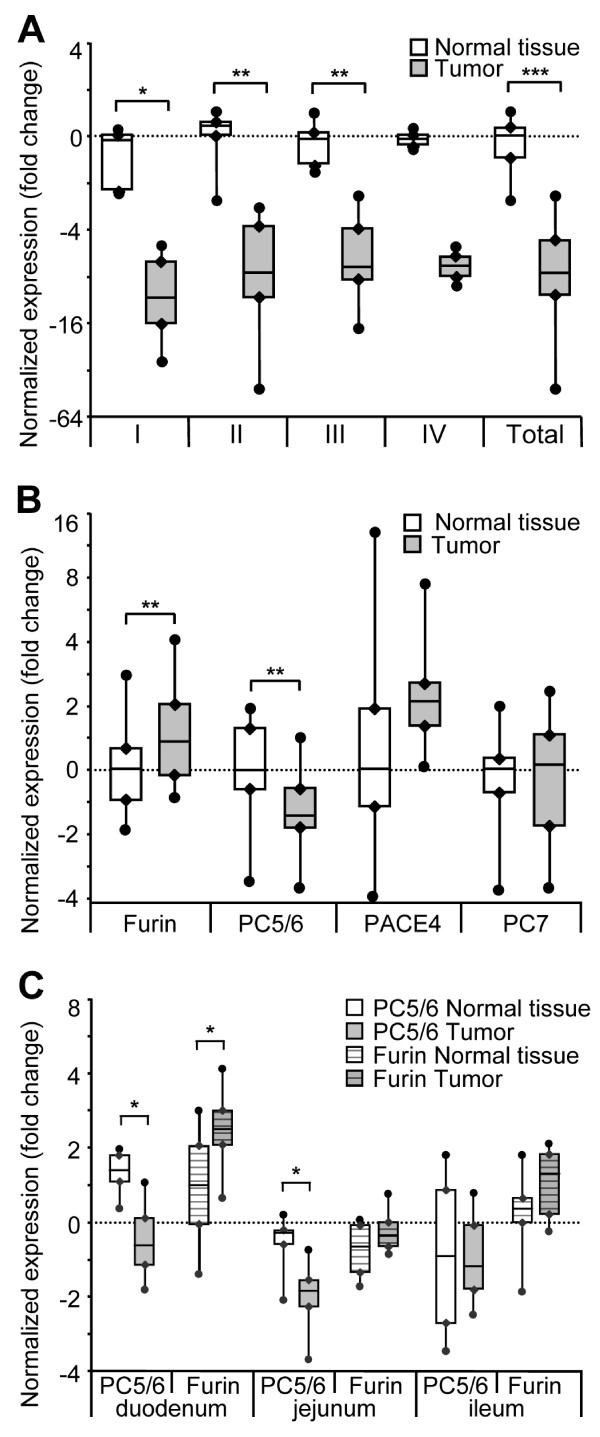
**Decreased expression of PC5/6 in intestinal tumors *versus *adjacent normal tissues**. (A) RNA samples from human colonic adenocarcinomas (stage I, II, III or IV) and their adjacent normal tissues were submitted to QPCR analysis (n = 6, 7, 7 and 2 for stages I, II, III and IV, respectively). (B) In each small intestine section (duodenum, jejunum and ileum) from 3 *Apc*^*Min*/+ ^mice, 2 tumors and their adjacent normal tissue (6 couples/section) were dissected and assessed for the expression levels of furin, PC5/6, PACE4 and PC7 by QPCR. Normalized expression values are shown for the 18 samples of normal tissues and 18 samples of tumors. (C) Expression of PC5/6 and furin in tumors was also analyzed by intestinal section. All mRNA levels in tumors were normalized to their respective normal tissue expression and have been log_2 _transformed, with the median of the total 18 samples set to 0. *, *P *< 0.05; **, *P *< 0.005; ***, *P *< 5.10^-11 ^(Student's *t *test).

Mining cancer gene expression database  revealed that PC5/6 expression was significantly reduced in 7 out of 10 tumor types (*P *< 0.0001); [see Additional file 1: figure S1]. Since PC5/6 expression is highest in the adult small intestine [29,31], and as no data were available for intestinal cancers, PC5/6 mRNA levels were analyzed by QPCR in 22 human colon tumors at stages I, II, III or IV and compared to those of their match-paired normal adjacent tissue (Figure 1A). PC5/6 expression was on average ~ 7.6-fold lower in these human tumors. To assess whether PC5/6 was similarly regulated in mouse, we used the *Apc*^*Min*/+ ^mice, which spontaneously develop numerous tumors in the small intestine due to the heterozygote mutation *Min *in the *Apc *gene. This mutation was originally discovered in patients suffering from familial adenomatous polyposis and frequently found in sporadic colorectal cancers [38,39]. Apc^Min/+^-induced tumors in the mouse small intestine constitute a good model for colonic tumorigenesis in human. We first quantified the expression levels of furin, PC5/6, PACE4 and PC7, which transit through the constitutive secretory pathway and cleave their substrates after basic residues [2]. While PACE4 and PC7 did not show any significant change, furin and PC5/6 mRNA levels were on average ~ 1.5-fold higher (*P *= 0.003) and lower (*P *= 0.0008), respectively (Figure 1B). Closer analysis of the duodenum-, jejunum- and ileum-associated tumors *versus *their adjacent normal tissues revealed a 1.9-, 1.2- and 1.4-fold higher furin levels, respectively, and a 2-, 1.7- and 1.1-fold lower PC5/6 expression, respectively (Figure 1C). Using specific primers, we showed that this lower level primarily affected PC5/6B transcripts [see Additional file 22: figure S2], which dominate in intestine [31]. The above data thus indicated that PC5/6 is down-regulated in many tumor types, including intestinal ones, and that in the latter furin undergoes an opposite up-regulation. Both PC5/6 and furin exhibited the greatest changes in the duodenum. These data prompted us to verify if intestinal tumorigenesis was favored in absence of PC5/6.

### Conditional inactivation of *Pcsk5 *in enterocytes

**Figure 2:**
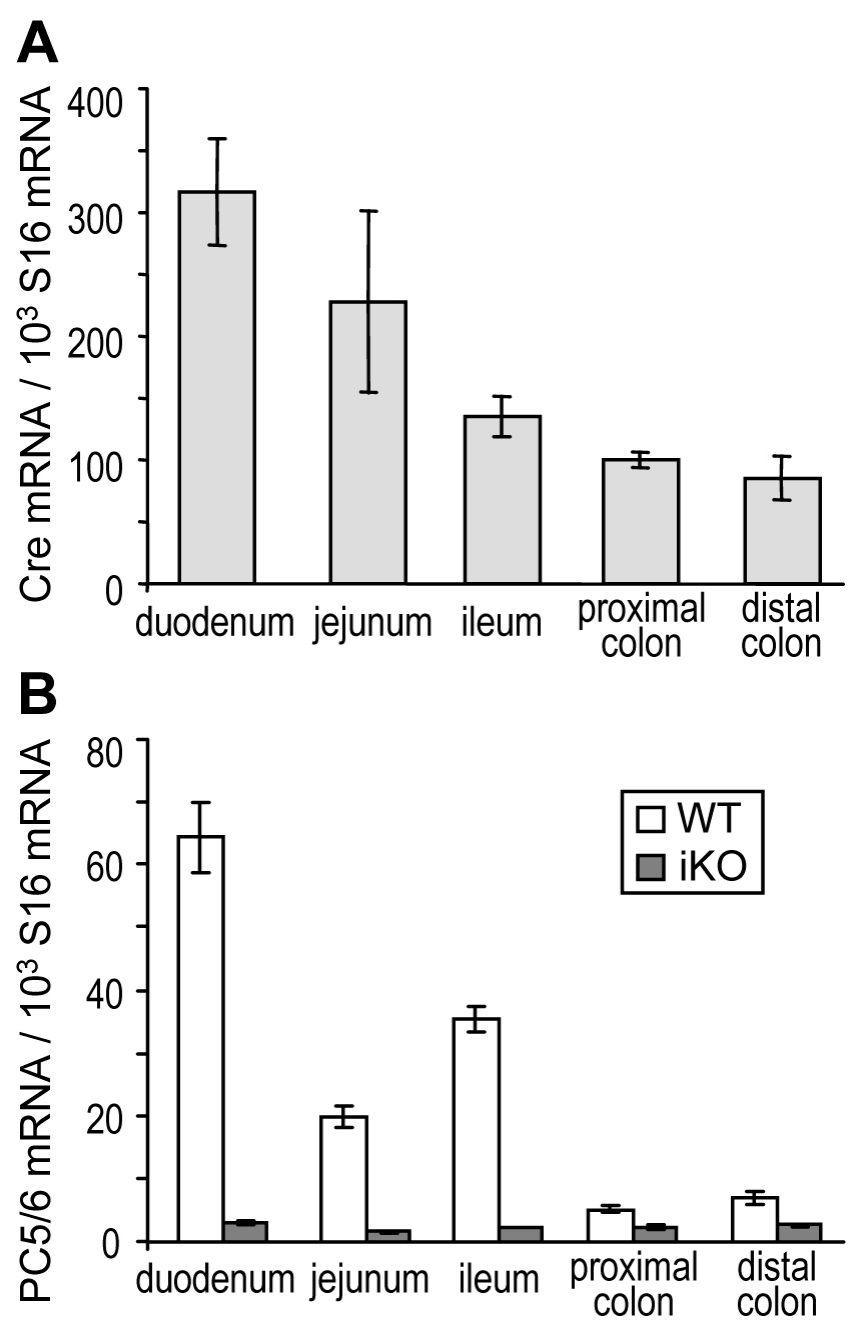
**Efficient inactivation of *Pcsk5 *in iKO mice**. (A) Cre expression was assessed in intestinal segments from 3 iKO mice. Expression values were normalized to that of S16 mRNA. (B) PC5/6 expression was quantified in each intestinal segment from 3 WT and 3 iKO mice and normalized to that of S16. Error bars represent SEM.

**Figure 3:**
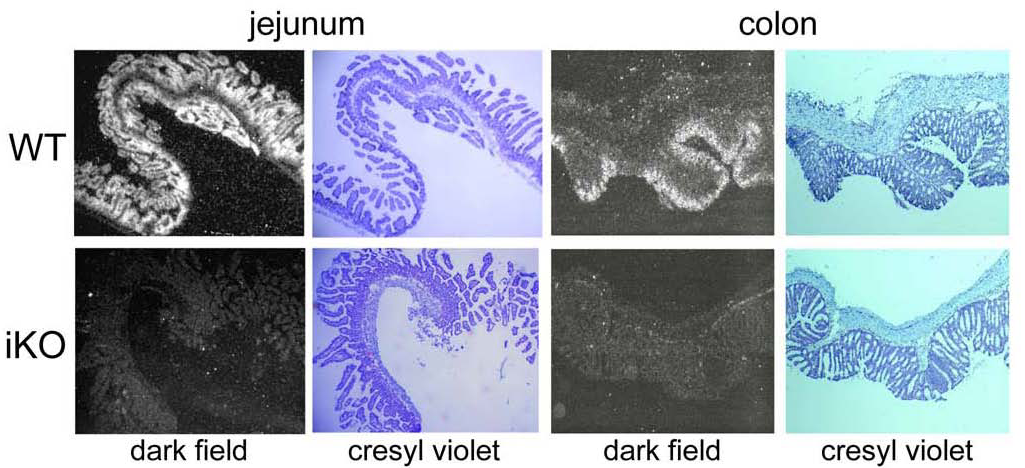
**Detection of PC5/6 transcripts in WT and iKO intestine by *in situ *hybridization**. Cryosections were hybridized with a PC5/6-specific probe, stained with cresyl violet and dipped in an autoradiography emulsion. The extent of ^35^S labeling was visualized on dark field.

To explore the *in vivo *role of PC5/6 in intestinal tumor formation, we specifically inactivated its gene in enterocytes using a *loxP*/Cre system. *Pcsk5*^*flox*/*flox *^mice were bred to Tg(Vil-cre) mice that expressed the Cre recombinase under the direction of the villin promoter, specifically expressed in enterocytes [40]. *Pcsk5*^*flox*/*flox *^mice carrying one copy of the transgene (iKO; Tg^+/0^) or none (WT; Tg^0/0^) were generated. To verify that the presence of the transgene resulted in an efficient inactivation of *Pcsk5 *in enterocytes, we analyzed PC5/6 mRNA levels using QPCR and *in situ *hybridization in 3 mice of each genotype. Duodenum, jejunum, ileum and colon sections were dissected for further RNA extraction and tissue sectioning. Cre expression under the villin promoter in iKO mice was highest in duodenum and progressively diminished along the intestinal tract to reach ~ 25% of the duodenum level in the distal colon (Figure 2A). In WT mice, PC5/6 expression is elevated in the small intestine, especially in the duodenum, as compared to colon (Figure 2B). Indicative of the Cre efficiency all along the intestine, the absolute numbers of PC5/6 mRNA remaining in all sections of iKO intestine were very similar: 1.6 to 3.1 PC5/6 mRNA/1000 S16 mRNA. Furthermore, *in situ *hybridization with a PC5/6 cRNA probe confirmed that PC5/6 transcripts were strongly reduced in iKO intestinal enterocytes (Figure 3). The low residual expression observed by QPCR (Figure 2B) and *in situ *hybridization labeling suggest that in the small intestine PC5/6 is mainly expressed in enterocytes, but to a much less extent expressed in other cell types all along the intestine. Finally, the morphology and proliferation of enterocytes was assessed by immunohistochemistry. No gross malformation was observed and labeling with PCNA, a marker for proliferation, was not significantly different between the two genotypes [see Additional file 3: figure S3].

### PC5/6 deficiency has a significant impact on Min mutation-induced tumorigenesis in the duodenum

**Figure 4:**
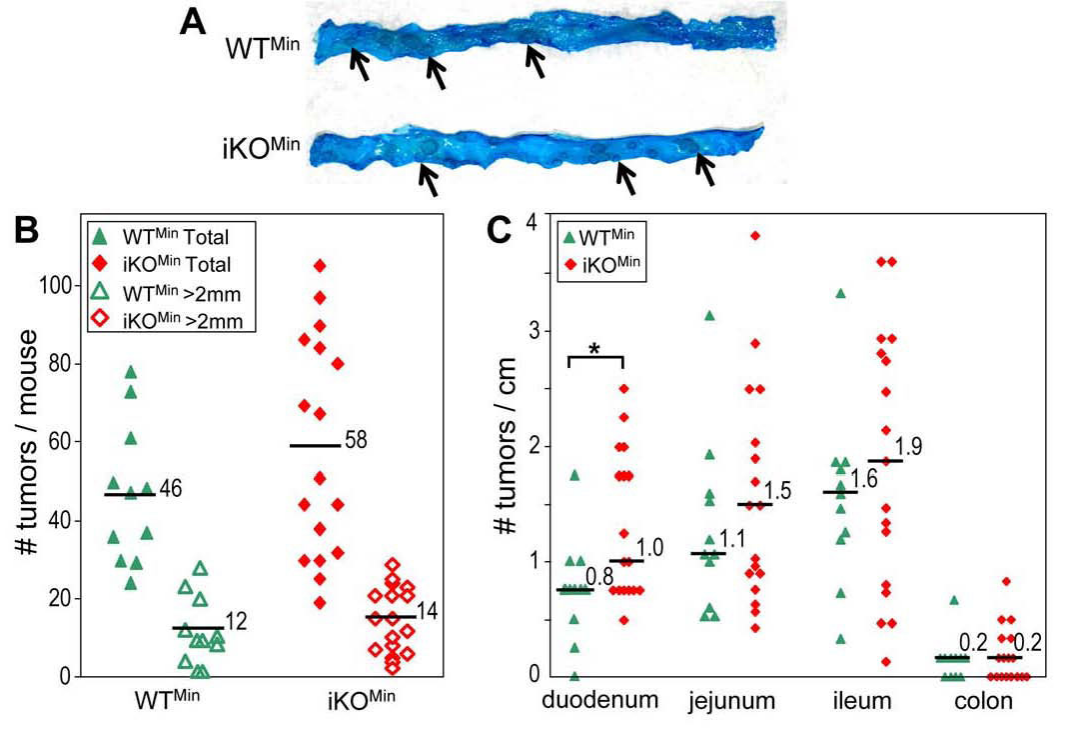
**Intestinal tumor formation in WT^Min ^and iKO^Min ^mice**. (A) Representative sections of WT^Min ^and iKO^Min ^ileum stained with methylene blue. Arrows point at visualized tumors. (B) Total tumor numbers and large tumor (> 2 mm) numbers in WT^Min ^and iKO^Min ^intestine of 4 month-old WT^Min ^(n = 11) and iKO^Min ^mice (n = 17). (C) Numbers of tumors per cm of duodenum, jejunum, ileum or colon in the above mice are shown. *, *P *< 0.05 (Student's *t *test)

Intercrossing of [*Pcsk5*^*flox*/*flox *^Tg(Vil-cre)^+/0^] with [*Pcsk5*^*flox*/*flox *^*Apc*^*Min*/+^] generates 25% mice that carry only the *Min *mutation (WT^Min^), and exhibit normal levels of PC5/6 in intestine. Another 25% of these mice carry both the *Min *mutation and the Cre transgene (iKO^Min^), and lack PC5/6 expression in enterocytes. Duodenum, jejunum and ileum from 11 WT^Min ^mice and 17 iKO^Min ^mice were dissected out, opened longitudinally and stained with methylene blue (Figure 4A). All the tumors, including those exceeding 2 mm in diameter, were counted along the entire section of each tissue. The average tumor density (tumors/cm) in the duodenum of iKO^Min ^mice was significantly higher than that in WT^Min ^mice (*P *= 0.01; Figure 4B). In iKO mice, the duodenum is the tissue in which the PC5/6 drop was the most drastic (Figure 2). However, although this trend was observed in other intestinal sections, it did not reach statistical significance, and the total number of tumors in iKO^Min ^mice, 58 *versus *46 in WT mice, was not significantly higher (Figure 4C). In addition, the numbers of large tumors (>2 mm; Figure 4C) were very similar in both cases. Overall, this analysis indicates that only in duodenum does the loss of PC5/6 significantly enhance intestinal tumorigenesis.

### PC5/6 deficiency shortens the half-life of *Apc*^*Min*/+ ^mice

**Figure 5:**
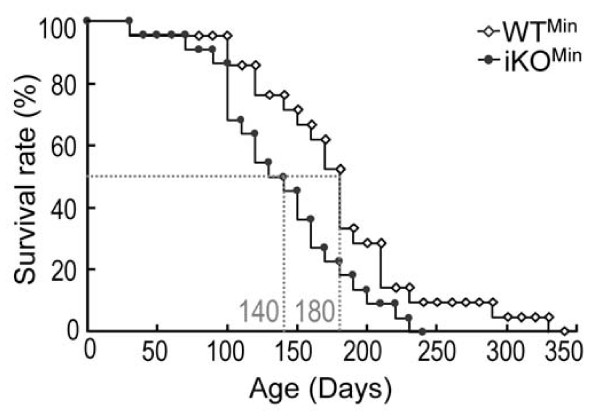
**Decreased survival of *Apc*^*Min*/+ ^mice in the absence of PC5/6**. Survival rates of WT^Min ^(n = 21) and iKO^Min ^(n = 22) mice were compared. *P *= 0.03 (Log-rank test)

*Apc *^*Min*/+ ^mice having a pure C57BL/6 background were reported to die by 120 days of age [38,39], likely due to severe chronic anemia [38]. In this study, WT^Min ^mice exhibited a longer half-life of 180 days, possibly due to their mixed background (*see ***Methods**). However, in the absence of intestinal PC5/6, this half-life was significantly shortened to 140 days (*P *= 0.03; Figure 5), suggesting that PC5/6 exerts a protective effect on these mice. *Apc*^*Min*/+ ^mice develop anemia with a severity that seems to depend on the density of intestinal adenomas [38]. Considering that iKO^Min ^mice had a trend for higher numbers of tumors, especially in the duodenum, premature death of iKO^Min ^mice could be the result of more severe chronic anemia [38], which could be exacerbated by multiple hemorrhages, as observed in the liver and subcutaneously in PC5/6 knockout mice [15]. In the future, it may be valuable to examine whether PC5/6 levels correlate with the survival rate, or intestinal bleeding/anemia of patients that suffer from colorectal carcinomas.

## Discussion

The use of general PC-inhibitors such as α1-PDX or pro-furin revealed that PC-inhibition decrease tumorigenesis and metastasis in nude mice [9,12,20,26], but enhance metastasis in immunosuppressed newborn rats [43]. This is probably due to the ability of overexpressed PC-inhibitors to block the activity of more than one convertase [44], which may exert opposite regulating effects and modulate multiple processes. Thus, mice lacking a specific convertase should represent a more powerful tool to assess the specific function of a single convertase. Of all the PC knockout mice, those lacking furin [45] and PC5/6 [15,16] exhibit a fully penetrant embryonic lethal phenotype, precluding their use in adult mouse studies. Tissue-specific knockouts thus provide a potential approach to test their effect in cancer/metastasis. So far, the *in vivo *role of a specific PC in tumorigenesis was only investigated in mice lacking furin in salivary glands among other tissues [46]. In these mice, the simultaneous inactivation of furin and overexpression of the *PLAG1 *transcription factor, which induced the formation of adenomas in salivary glands, showed that the absence of furin delayed tumorigenesis [46], suggesting a pro-tumorigenic effect of furin.

The present study is the first attempt to assess the role of PC5/6 in cancer development using knockout mice. The impact of PC5/6 has been analyzed here exclusively *in vivo*, using the *Apc*^*Min*/+ ^intestinal tumorigenesis model. We first evaluated PC5/6 mRNA levels in intestinal tumors *versus *normal tissue obtained from colon cancer patients (Figure 1A) or *Apc*^*Min*/+ ^mice (Figure 1B and 1C), and showed that PC5/6 is systematically down-regulated in intestinal tumors. To probe the role of PC5/6 in tumorigenesis, we compared the number and size of intestinal tumors in *Apc*^*Min*/+ ^mice lacking or not PC5/6 (Figure 4). The data showed a trend for an enhanced tumorigenesis in PC5/6-deficient mice, reaching significance only in the duodenum (Figure 4B) where PC5/6 is primarily expressed (Figure 2A), suggesting that it may exert specific functions therein. This result was unexpected in view of the reported reduced tumorigenesis by general PC-inhibitors [18,20-22].

Could PC5/6 specifically process a tumor-suppressor or inactivate a tumorigenic factor, and hence act in an opposite fashion to other basic aa-specific PCs? Opposing functions can occur by cleavage of the same substrate at different sites, as illustrated by the ability of furin to activate the cell adhesion molecule N-cadherin and PC5/6 to inactivate it (Maret D. *et al*., *submitted*). In the duodenum, PC5/6 was only 1.7-fold less abundant than furin, while its ratio to furin was 3- to 10-fold lower in other segments of the intestine [see Additional file 44: figure S4]. Thus, tumorigenesis in the duodenum may depend on the balance between activation and/or inactivation of proteins by resident furin and PC5/6, respectively. In tumors of the duodenum, PC5/6 mRNA levels are ~ 7-fold lower than those of furin (Figure 1C). Thus, the pro-tumorigenic properties of furin [46] may in some cases overshadow the protective effect of PC5/6. We surmise that within the duodenum, furin may activate precursors implicated in epithelial to mesenchymal transition, involved in early tumorigenesis and invasion/metastasis [47], such as E-cadherin [48] and TGF-β [49], while PC5/6 may inhibit tumorigenesis, e.g., *via *inactivation of adhesion proteins such as N-cadherin (Maret D. *et al*., *submitted*), resulting in a lower number of tumors.

## Conclusion

Future studies aimed to identify the implicated substrates will require an extensive comparative analysis of *Apc*^*Min*/+^-induced tumors isolated from mice lacking PC5/6, furin or both in enterocytes. Whether the mechanism behind the shortened survival of *Apc*^*Min*/+ ^mice lacking PC5/6 (Figure 5) is due to more severe hemorrhages resulting from a greater vessel fragility induced by the loss of PC5/6 [15] would require a more detailed examination. Furthermore, the importance of specific PCs in the invasion/metastasis process, which is heavily regulated by adhesion molecules processed by PCs [17,27] is yet to be fully investigated in an appropriate *in vivo *model. Finally, this is the first report that emphasizes the opposite roles of furin and PC5/6 in tumorigenesis. Thus, recently proposed treatments aimed to reduce furin activity [9,18-27] should include careful monitoring of their effects on PC5/6 levels and/or activity.

## Abbreviations

aa: amino acid; α1-PDX: α1-antitrypsin Portland; *Apc*: adenomatous polyposis coli; iKO: intestinal knockout of the *Pcsk5 *gene; *Min*: multiple intestinal neoplasia; PC: Proprotein convertase; PCNA: proliferation cell nuclear antigen; *Pcsk5*: Proprotein convertase subtilisin/kexin type 5; QPCR: quantitative RT-PCR; Tg: transgene; WT: wild type.

## Competing interests

This work was supported by Canadian Institutes of Health Research grant # 44363, a Canada Chair # 201652, and a Strauss foundation grant. The authors declare that they have no competing interests.

## Authors' contributions

All authors read and approved the final manuscript.

XS carried out all the mouse analyses, tumor measurements and other experiments as well as the genotyping. RE generated the PC5/6 conditional knockout mice and helped in the analyses of their phenotypes, NGS participated in the design of the experiments, analysis of the data and writing of the manuscript, and AP was the major driver of the project implicated in all aspects of the research.

## Supplementary Material

Additional file 1 **Down-regulation of PC5/6 expression in various cancers**. Datasets were retrieved from ONCOMINE (a cancer microarray database and integrated data-mining platform) with a threshold of *P *< 0.0001. PC5/6 expression value in tumors was log_2 _transformed and normalized by that in the adjacent normal tissue. Click here for file

Additional file 2 **Decreased expression of PC5/6B, but not PC5/6A, in intestinal tumors *versus *adjacent normal tissues**. Specific primers were used for QPCR analysis of the two PC5/6 isoforms. Normal (N) and tumoral (T) expression of PC5A and PC5B was assessed by using isoform-specific primers. Error bars represent SEM and n = 6 for each intestine section. *, *P *< 0.05 for PC5/6B (Student's *t *test) Click here for file

Additional file 3 **Unaffected enterocyte proliferation in iKO mice**. Representative PCNA immunohistochemistry of WT and iKO jejunum sections is shown. Quantitative analysis was achieved by counting PCNA-positive nuclei in 3 random fields in duodenum, jejunun and ileum in 3 mice per genotype. Error bars represent SEM. Click here for file

Additional file 4 **Relative expression of PC5/6 and furin in WT intestine**. The PC5/6 and furin expression was assessed on each intestinal segment from 3 WT mice. The expression value was normalized to that of S16 mRNA. Error bars represent SEM. Click here for file
