# Supplementary material for: The proprotein convertase PC5/6 is protective against intestinal tumorigenesis: in vivo mouse model
Source: Mol Cancer. 2009 Sep 8;8:73. doi: 10.1186/1476-4598-8-73 (PMC2746178; doi:10.1186/1476-4598-8-73)
Supplement: Additional file 1 — Down-regulation of PC5/6 expression in various cancers. Datasets were retrieved from ONCOMINE (a cancer microarray database and integrated data-mining platform) with a threshold of P < 0.0001. PC5/6 expression value in tumors was log2 transformed and normalized by that in the adjacent normal tissue. [file 1476-4598-8-73-S1.pdf]

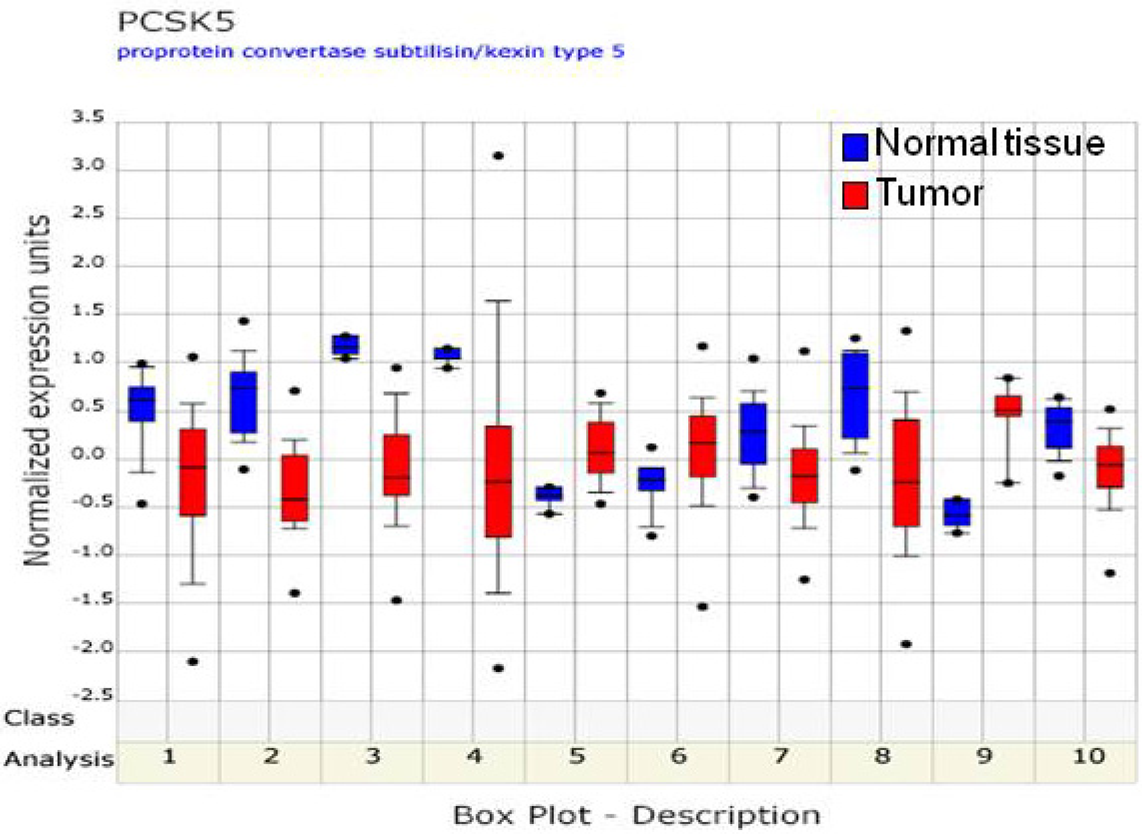

1. Bladder Carcinoma
2. Cutaneous Melanoma
3. Ovarian Endometrioid Adenocarcinoma
4. B-Cell Acute Lymphoblastic Leukemia
5. Anaplastic Oligoastrocytoma, Anaplastic Oligodendroglioma
6. Glioblastoma Multiforme
7. Prostate Carcinoma
8. Head and Neck Cancer
9. Pancreatic Ductal Adenocarcinoma
10. Head and Neck Squamous Cell Carcinoma
