# Supplementary figures and images for: The proprotein convertase PC5/6 is protective against intestinal tumorigenesis: in vivo mouse model
Source: Mol Cancer. 2009 Sep 8;8:73. doi: 10.1186/1476-4598-8-73 (PMC2746178; doi:10.1186/1476-4598-8-73)

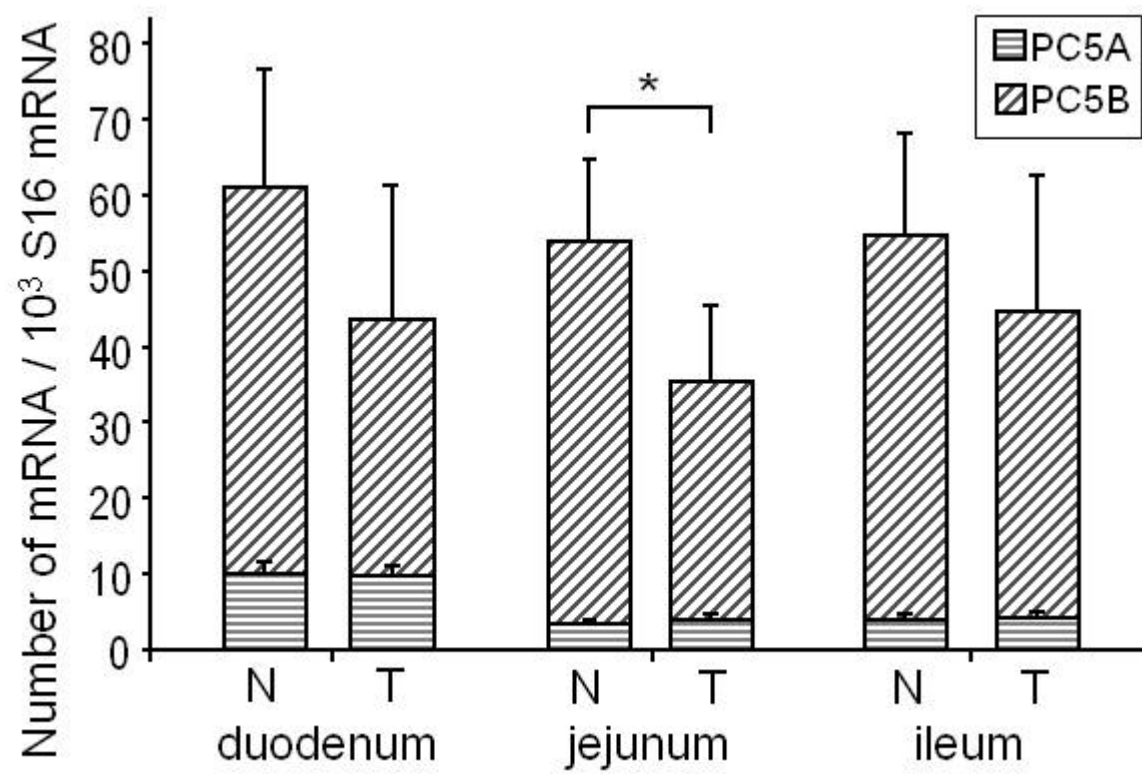

Supplement: Additional file 2 — Decreased expression of PC5/6B, but not PC5/6A, in intestinal tumors versus adjacent normal tissues. Specific primers were used for QPCR analysis of the two PC5/6 isoforms. Normal (N) and tumoral (T) expression of PC5A and PC5B was assessed by using isoform-specific primers. Error bars represent SEM and n = 6 for each intestine section. *, P < 0.05 for PC5/6B (Student's t test) [file 1476-4598-8-73-S2.pdf]

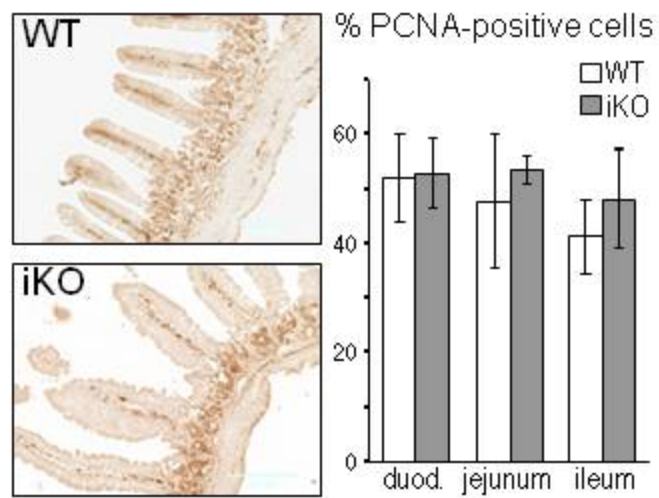

Supplement: Additional file 3 — Unaffected enterocyte proliferation in iKO mice. Representative PCNA immunohistochemistry of WT and iKO jejunum sections is shown. Quantitative analysis was achieved by counting PCNA-positive nuclei in 3 random fields in duodenum, jejunun and ileum in 3 mice per genotype. Error bars represent SEM. [file 1476-4598-8-73-S3.pdf]

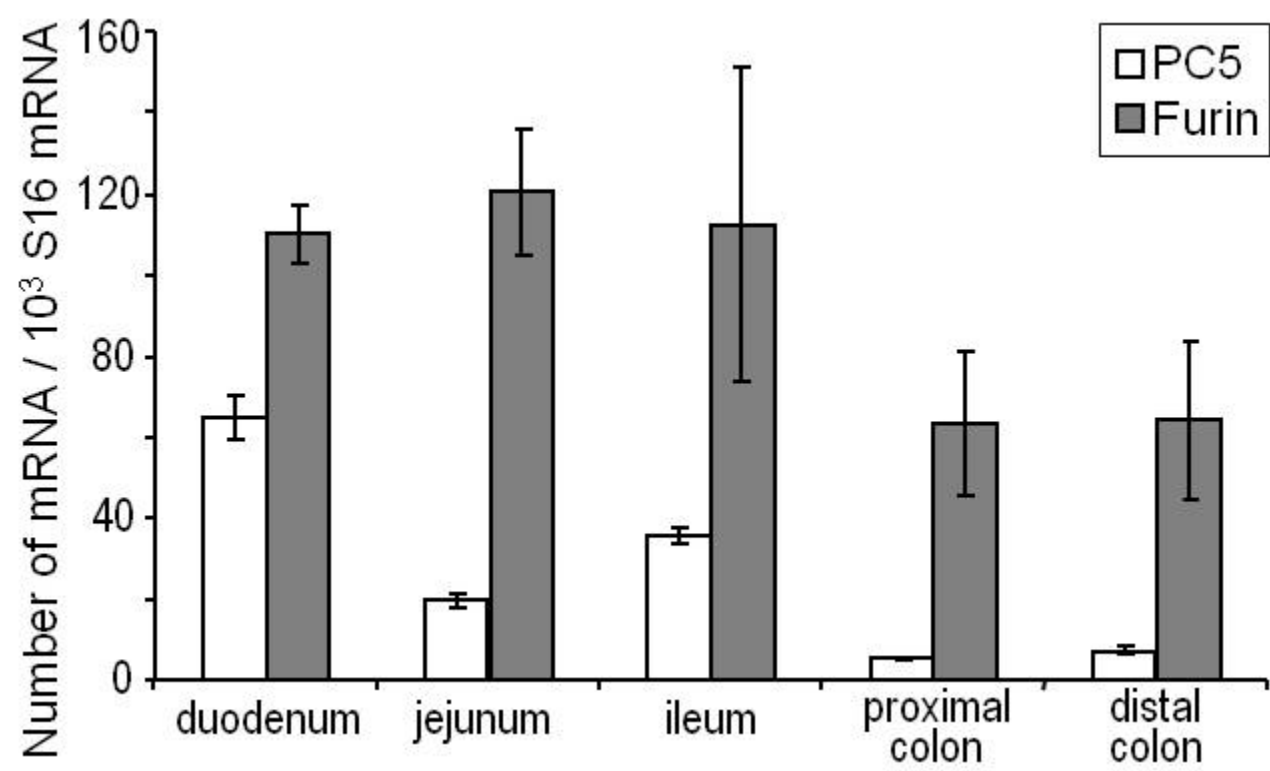

Supplement: Additional file 4 — Relative expression of PC5/6 and furin in WT intestine. The PC5/6 and furin expression was assessed on each intestinal segment from 3 WT mice. The expression value was normalized to that of S16 mRNA. Error bars represent SEM. [file 1476-4598-8-73-S4.pdf]
